# Polyclonal Expansion of NKG2C^+^ NK Cells in TAP-Deficient Patients

**DOI:** 10.3389/fimmu.2015.00507

**Published:** 2015-10-06

**Authors:** Vivien Béziat, Marwan Sleiman, Jodie P. Goodridge, Mari Kaarbø, Lisa L. Liu, Halvor Rollag, Hans-Gustaf Ljunggren, Jacques Zimmer, Karl-Johan Malmberg

**Affiliations:** ^1^Department of Medicine Huddinge, Center for Infectious Medicine, Karolinska Institutet, Stockholm, Sweden; ^2^Laboratory of Human Genetics of Infectious Diseases, Necker Branch, INSERM U1163, Paris, France; ^3^Imagine Institute, University Paris Descartes, Paris, France; ^4^Luxembourg Institute of Health, House of Biohealth, Esch-sur-Alzette, Luxembourg; ^5^Department of Cancer Immunology, Institute for Cancer Research, Oslo University Hospital, Oslo, Norway; ^6^The KG Jebsen Center for Cancer Immunotherapy, Institute of Clinical Medicine, University of Oslo, Oslo, Norway; ^7^Department of Microbiology, Oslo University Hospital, Oslo, Norway

**Keywords:** natural killer cells, transporter associated with antigen processing, adaptive immunity, cytomegalovirus infections, killer cell immunoglobulin-like receptor

## Abstract

Adaptive natural killer (NK) cell responses to human cytomegalovirus infection are characterized by the expansion of NKG2C^+^ NK cells expressing self-specific inhibitory killer-cell immunoglobulin-like receptors (KIRs). Here, we set out to study the HLA class I dependency of such NKG2C^+^ NK cell expansions. We demonstrate the expansion of NKG2C^+^ NK cells in patients with transporter associated with antigen presentation (TAP) deficiency, who express less than 10% of normal HLA class I levels. In contrast to normal individuals, expanded NKG2C^+^ NK cell populations in TAP-deficient patients display a polyclonal KIR profile and remain hyporesponsive to HLA class I-negative target cells. Nonetheless, agonistic stimulation of NKG2C on NK cells from TAP-deficient patients yielded significant responses in terms of degranulation and cytokine production. Thus, while interactions with self-HLA class I molecules likely shape the KIR repertoire of expanding NKG2C^+^ NK cells during adaptive NK cell responses in normal individuals, they are not a prerequisite for NKG2C^+^ NK cell expansions to occur. The emergence of NKG2C-responsive adaptive NK cells in TAP-deficient patients may contribute to antiviral immunity and potentially explain these patients’ low incidence of severe viral infections.

## Introduction

Considerable lines of evidence indicate that human and mouse natural killer (NK) cells can undergo a phase of selective expansion in response to viral challenges (1, 2). In humans, such “adaptive” NK cell responses are typically linked to cytomegalovirus (CMV) infection, in isolation or in the context of other viral infections (3–9). Hallmarks of this response include selective expansion of NKG2C^+^ NK cells with expression of self-HLA class I-specific killer-cell immunoglobulin-like receptors (KIRs) (3, 4, 10). Such expanded cells are terminally differentiated and functionally reprogrammed involving epigenetic mechanisms (11). Although the molecular drivers of the expansion of specific NK cell subsets associated with CMV infection/reactivation are largely unknown, self-specific KIRs have been shown to provide a survival advantage during the expansion phase (12). Hence, it has been suggested that NK cell interaction with HLA class I molecules is important for shaping adaptive NK cell responses (13). However, the role of self-MHC class I interactions in driving adaptive NK cell responses to viral infections is challenged by findings in β_2_m-deficient mice that display expansion of Ly49H^+^ adaptive NK cells at levels similar to wild-type mice (14) and are fully capable of controlling mouse CMV (15, 16).

To specifically address the role of HLA class I molecules in context of human NK cell expansions, we applied a recently described algorithm for tracing adaptive-like NK cell responses based on cellular differentiation states (17) and applied this in patients with transporter associated with antigen presentation (TAP) deficiency. Human TAP deficiency is a rare genetic immunodeficiency (18) and results in less than 10% of normal HLA class I expression. Conceivably, NK cells from TAP-deficient patients are not educated by self HLA class I, resulting in hypofunctionality of the NK cells in terms of low levels of natural cytotoxicity against HLA class I low targets (17, 19–21). A similar phenotype was recently reported in two β2m-deficient siblings (22). Yet, NK cells from TAP-deficient patients display normal levels of activating receptors and an intact cytotoxic machinery (20). Similar to the small population of non-educated NK cells in normal individuals, NK cells from TAP-deficient patients readily perform antibody-dependent cellular cytotoxicity (ADCC) (21). In these respects, the functionality of NK cells from TAP-deficient individuals resembles that of non-educated NK cells in MHC class I-deficient mice lacking either TAP or β_2_-microglobulin (23–25).

We here report the expansion of differentiated, at least partially functional, NKG2C^+^ NK cells in TAP-deficient patients. Notably, in contrast to healthy donors, the expanded NK cell populations observed in the patients were polyclonal with respect to KIR expression; hence, they were not skewed toward expression of a self-specific KIR. Thus, interactions with self-HLA class I skew the NK cell repertoire with respect to KIR expression during the expansion phase of the NK cells, but this skewing is not, *per se*, a prerequisite for the development of an adaptive NK cell response.

## Materials and Methods

### Patients

This study was approved by the regional ethics committee in Stockholm, Sweden, and by the National Research Ethics Committee of Luxembourg (CNER, 201109/05). All individuals included gave written informed consent according to the Declaration of Helsinki. Peripheral blood mononuclear cells (PBMCs) were separated from buffy coats by density gravity centrifugation (Ficoll-Paque; GE Healthcare) and frozen in FCS with 10% DMSO until use.

### Flow cytometry

Killer-cell immunoglobulin-like receptor repertoire staining, functional assays, and data analysis were performed as described in detail elsewhere (3). In brief, freshly thawed PBMCs of patient and controls were stained with the following monoclonal antibodies: CD3-PE.Cy5, CD14-PE.Cy5, CD19-PE.Cy5, CD56-ECD, ILT2-PE, CD161-PE, CD7-PE-Cy7, and NKG2A-APC.AF750, all from Beckman Coulter; NKG2C-PE or biotin from R&D Systems; PLZF-PE, NKp30-APC, and NKp46-PE from Becton Dickinson; FcϵRγ1-FITC from Millipore; and CD57-PB from Biolegend. Dead cells were excluded by using the aqua live/dead kit (Invitrogen).

### Functional assays

For functional experiments, PBMCs were thawed, rested overnight, and then mixed with K562 cells or RAJI cells or P815 cells at a ratio of 10:1 in U-bottomed 96-well plates and incubated for 6 h at 37°C and 5% CO_2_. For redirected ADCC assays, P815 cells were incubated with 5 μg/mL of the indicated anti-NKG2C. For ADCC experiments, RAJI cells were incubated with 1 μg/mL of rituximab (anti-CD20). Preceding the assay, CD107a-FITC (H4A3) was added together with brefeldin (Golgi Plug, 1/1000, Becton Dickinson) and monensin (Golgi Stop, 1/1500, Becton Dickinson). For polyfunctional assays, cells were permeabilized (Fixation & Permeabilization Buffers, eBioscience) and then stained with intracellular IFNγ-AF700 (Becton Dickinson) and TNFα-eF450 (eBioscience) and analyzed by flow cytometry. Pie charts were generated using the Spice software (26).

### CMV infection and HLA-I expression analysis

The three fibroblast cell lines used for this study (STF5-169, healthy donor; STF1-169, TAP-2 deficient; BRE-169, TAP-1 deficient) were immortalized with the vector pl169. The cells were synchronized by contact inhibition and plated at a density of 10^6^ cells/75 cm^2^ to induce cell cycle progression. At the same time, the cells were infected with AD169 CMV strain at a multiplicity of infection of 5 plaque-forming units per cell or the equivalent of mock media. Forty-eight hours post infection, cells were stained for MHC class I expression with a biotinylated anti-pan HLA class I mAb (W6/32) or anti-HLA-E mAb (3D12), detected with Streptavidin-PE, and fixed. After permeabilization, cells were monitored for the expression of the immediate early antigen 1 (IE-1) using anti-CMV IE-1-AF488 (Millipore).

## Results

### Expansion of NKG2C^+^ NK cells in patients with TAP deficiency

**Figure 1:**
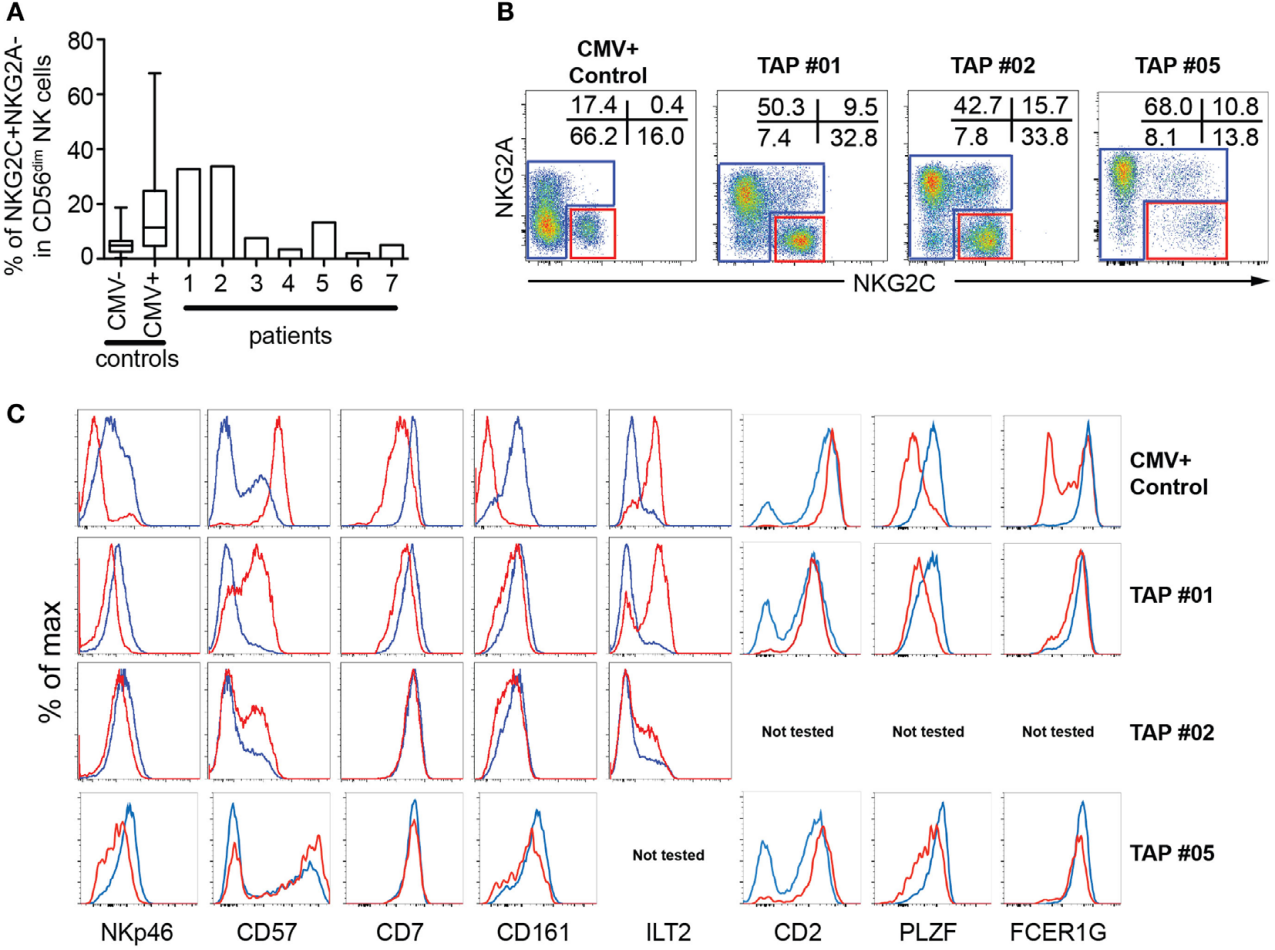
**Phenotypic characterization of NKG2C^+^ NK cells in TAP-deficient individuals**. **(A)** Size of the NKG2C^+^NKG2A^−^ subset in PBMCs from seven TAP-deficient donors compared to CMV^−^ and CMV^+^ healthy controls. **(B, C)** Gating strategy and phenotype of NKG2C^+^NKG2A^−^ (red lines) compared to conventional CD56^dim^ NK cells (blue lines) after gating on live CD3^−^CD4^−^CD14^−^CD19^−^CD7^+^CD56^dim/−^ cells from three TAP-deficient donors compared to one healthy control.

To study the occurrence of expanded NK cell populations in patients lacking expression of normal levels of HLA class I molecules, we assessed the expression of NKG2C on NK cells from seven previously described TAP-deficient patients for which sufficient numbers of PBMCs were available for detailed phenotypic and functional characterization (27–31). Two of these patients (TAP#01 and TAP#02) displayed increased frequencies of NKG2A^−^NKG2C^+^ NK cells corresponding to those observed in CMV^+^ healthy donors (Figure 1). One patient (TAP#05) displayed an expanded but yet smaller population of NKG2A^−^NKG2C^+^ NK cells. The remaining four patients had frequencies of NKG2A^−^NKG2C^+^ NK cells in the range of those observed in CMV^−^ healthy donors (3).

The expanded NKG2C^+^ NK cells in the TAP-deficient patients displayed phenotypic characteristics typical of terminal differentiation, including low expression of NKp46, and PLZF and increased expression of CD2, CD57, and LIR-1 (Figure 1). The expression of these markers appeared somewhat less pronounced in the patients compared to those commonly observed in CMV^+^ healthy individuals. For example, the effect on CD7 was marginal and no loss of FcϵRγ1 was observed in any of the three patients analyzed (Figure 1). Although this could be a coincidental finding in these patients, possibly the mild differentiation phenotype represented a less powerful or incomplete differentiation process of NKG2C^+^ NK cells in TAP-deficient patients. In support of the latter interpretation, there is evidence suggesting that the steady-state differentiation is impeded in TAP-deficient patients with high expression of NKG2A and low frequencies of CD57^+^ NK cells (30).

### NKG2C^+^ NK cells from TAP-deficient patients display polyclonal KIR repertoires

**Figure 2:**
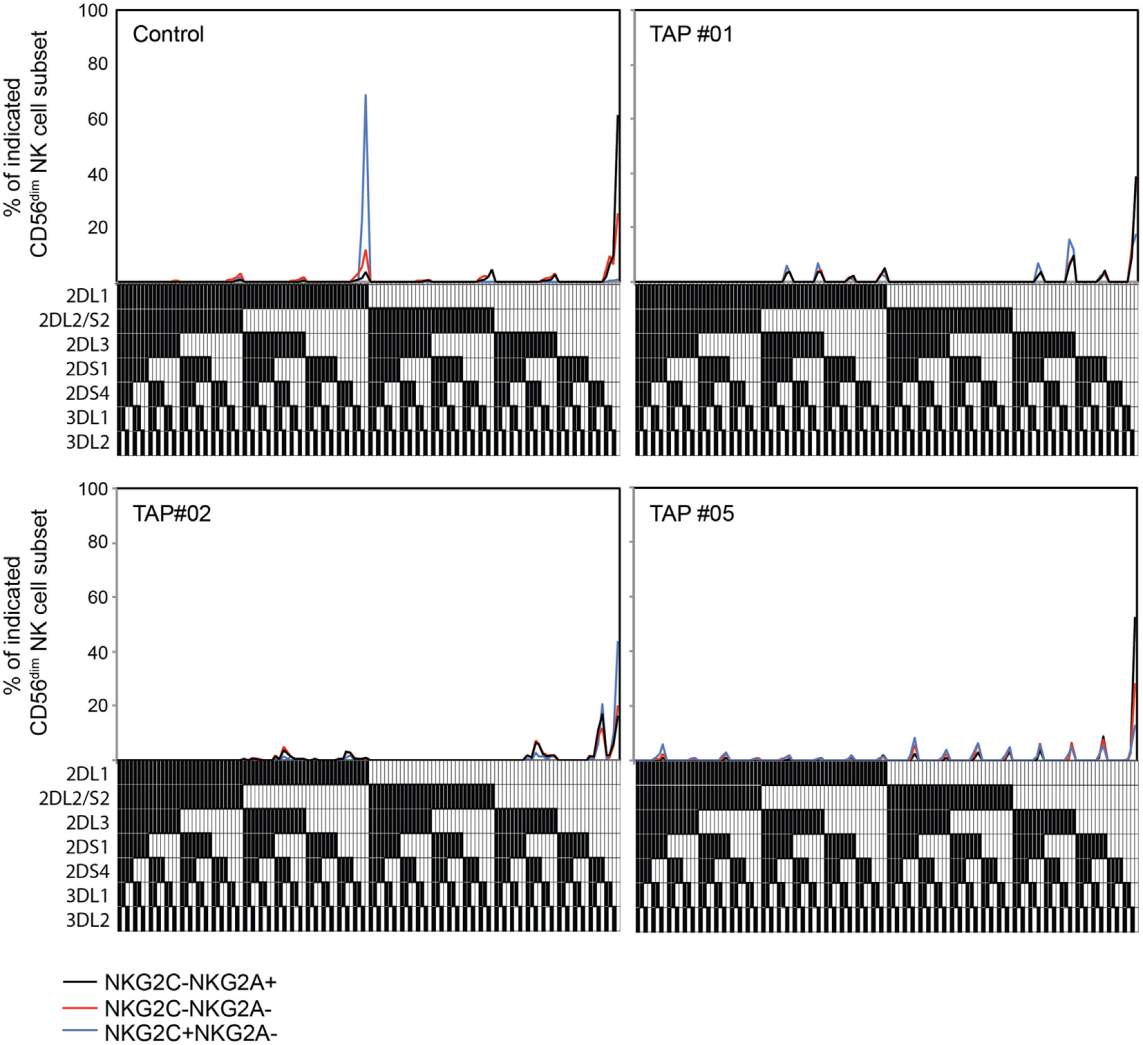
**Adaptive NK cells from TAP-deficient patients display polyclonal KIR repertoires**. Frequencies of NK cell subsets expressing seven KIRs analyzed, and the 128 possible combinations thereof, in one healthy control and three TAP-deficient individuals. The presence of one KIR in a combination is represented by a color code below the graph. The analysis is displayed for NKG2A^+^NKG2C^−^ (black lines), NKG2A^−^NKG2C^−^ (red lines), and NKG2A^−^NKG2C^+^ (blue lines) NK cell subsets. Patients TAP#1 and #5 are KIR haplotype B/X and patient TAP#2 is haplotype A/A.

In healthy individuals, the expansion of NKG2C^+^ NK cells is more or less confined to subsets expressing at least one self-specific KIR (3, 4, 10). This biased expansion was recently attributed to an increased resistance to starvation-induced apoptosis in educated NK cells (12). Thus, it is possible that educated and uneducated NK cell subsets respond equally well to NKG2C ligation, but only those that express self-specific KIR tolerate the stress of cytokine withdrawal after an initial inflammatory response to acute infection, resulting in accumulation of self-specific KIR-expressing NK cells. When examining the KIR-expression profiles in TAP-deficient patients, no skewing of KIR expression among differentiated NKG2C^+^ NK cells was observed (Figure 2). These results show that the expression of a self-KIR does not provide any selective benefit in conditions of low HLA class I expression.

### CMV infection does not interfere with HLA-E expression in TAP-deficient cells

**Figure 3:**
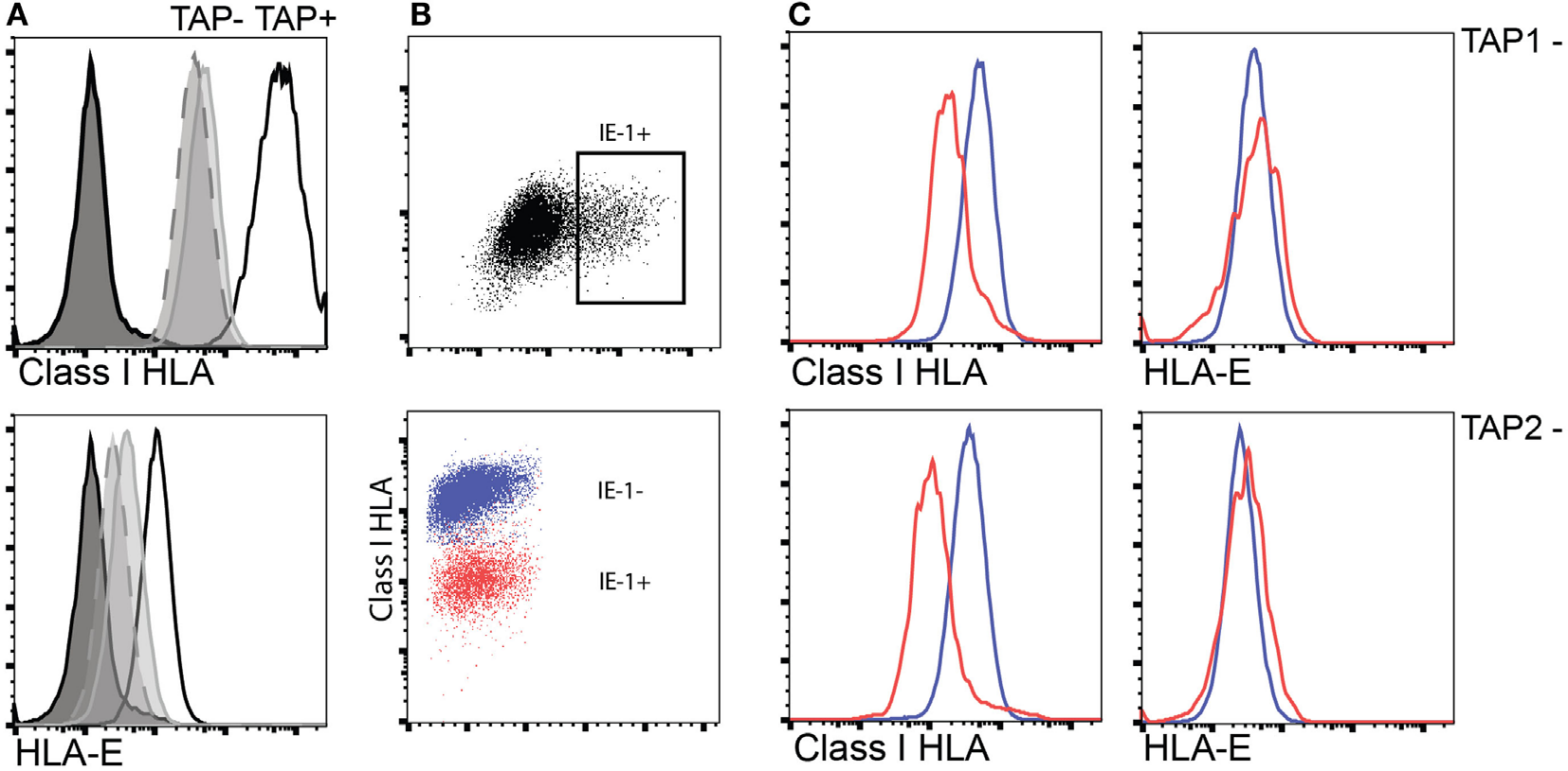
**Interference with HLA class I surface expression upon CMV infection does not extend to HLA-E in TAP-deficient fibroblast cell lines**. **(A)** Expression of total surface HLA class I (upper) and HLA-E (lower) in either TAP-1 (BRE-169, dashed) or TAP-2 (STF1-169, solid gray)-deficient fibroblast cell lines versus a TAP-expressing control fibroblast cell line (STF5-169, bold). **(B,C)** Expression of total HLA class I and HLA-E following infection of TAP-deficient fibroblast cell lines as indicated by intracellular expression of the CMV IE-1 antigen (upper scatter plot).

In TAP-deficient individuals, as mentioned, total HLA class I is reduced to approximately 10%. HLA-E expression is largely dependent on TAP-dependent mediated loading of leader-sequence peptides constituting the main cargo of HLA-E peptide content in normal TAP-expressing cells (32). Despite this, HLA-E expression appears to be less reduced (expression levels 30–50%) compared to total HLA class I in human TAP deficiency (30). To examine how the level of total HLA class I and HLA-E in TAP-deficient cells is affected by CMV infection, we established an *in vitro* infection model using fibroblasts derived from two TAP-deficient patients. Upon CMV infection, TAP-deficient fibroblasts manifested a further reduction of total HLA class I expression, whereas HLA-E expression remained intact compared to non-infected fibroblasts (Figure 3). Thus, TAP-deficient fibroblasts retained their HLA-E expression levels (i.e., 30–50% of normal levels) upon CMV infection. This outcome suggests that CMV could still induce NK cell activation via HLA-E-mediated triggering of NKG2C receptors.

### NKG2C is functional in TAP-deficient patients

**Figure 4:**
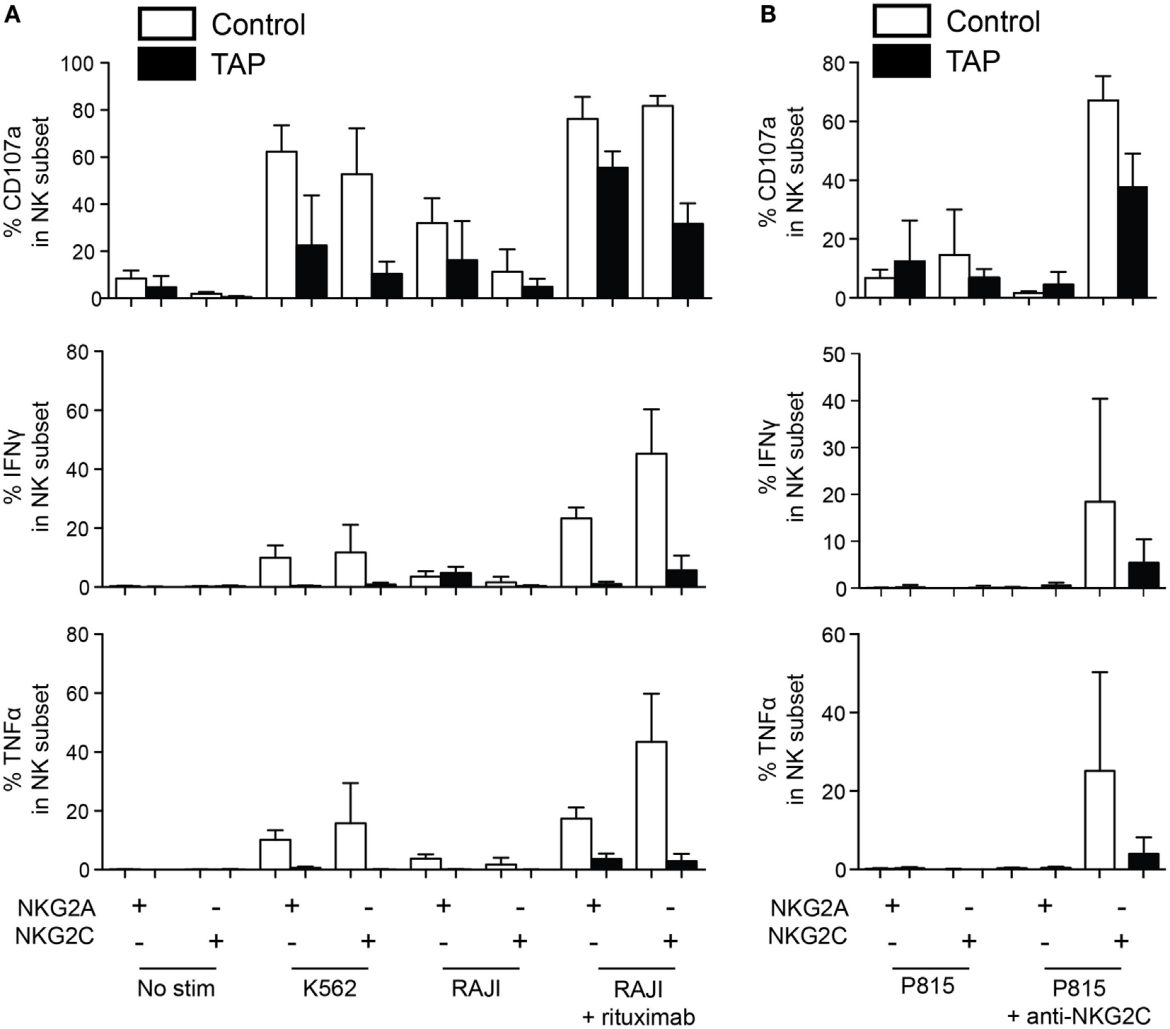
**NKG2C is functional in TAP-deficient adaptive NK cells**. **(A)** NK cells from healthy donors (average of four donors) and three TAP-deficient patients (TAP#01, TAP#02, and TAP#05) were stimulated with the indicated targets. RAJI cells were coated with anti-CD20 (rituximab, 1 μg/mL). NKG2C^+^NKG2A^−^ and NKG2C^−^NKG2A^+^ NK cell subsets were monitored for degranulation (CD107a) and cytokine production (IFN-γ and TNF-α). **(B)** Redirected ADCC assay using agonistic mAb against NKG2C. Degranulation (CD107a, top panel), IFN-γ (middle panel), and TNF-α (bottom panel) responses by the NKG2C^+^NKG2A^−^ and NKG2C^−^NKG2A^+^ NK cell subsets are displayed. The mean and standard deviation of four representative healthy controls tested simultaneously with the three TAP-deficient patients is shown.

It has been well documented that NK cells are hyporesponsive in TAP-deficient patients (17, 19–21), potentially due to inadequate HLA class I-mediated education. Therefore, we next set out to assess whether this hyporesponsiveness extended also to adaptive NK cells in these patients. Accordingly, we stimulated NK cells with K562 cells, RAJI cells alone, or RAJI cells coated with anti-CD20 (rituximab) and monitored polyfunctional responses in NKG2A^+^NKG2C^−^ and NKG2A^−^NKG2C^+^ NK cell subsets (Figure 4A). Although responses of NK cells from the three TAP patients with evidence of polyclonal NK cell expansions differed somewhat, the patterns were clearly distinct from those of NK cells from normal donors. Both conventional and expanded NK cells from TAP-deficient patients were generally hyporesponsive to K562 stimulation and produced cytokines, albeit at low levels, in response to any stimulation. Notably, TAP-deficient NK cells responded to ADCC, in line with the ability of CD16 ligation to partly overcome the need for education (33) (Figure 4A).

To more specifically address the functionality of the NKG2C pathway, NK cells were stimulated with P815 cells coated with an agonistic antibody against NKG2C (Figure 4B). As previously reported (34), resting NK cells from healthy donors responded strongly to ligation of NKG2C alone, without the need for ligation of additional coactivation receptors. Notably, also TAP-deficient NK cells responded to NKG2C ligation at levels comparable to those seen in response to ADCC stimulation. Again the response was largely restricted to degranulation albeit weak levels of IFN-γ and TNF were noted (Figure 4B). As expected, no response to ligation of NKG2C could be observed in NKG2A^+^NKG2C^−^ NK cells. Thus, although NK cells from TAP-deficient patients are hypofunctional and fail to respond to HLA class I-negative target cells, they still do respond, albeit at lower levels, to antibody-coated targets and direct ligation of the NKG2C receptor.

## Discussion

Human TAP deficiency is associated with severe bacterial lung infections and skin ulcers with chronic granulomatous inflammation. However, they rarely present with life-threatening viral infections and CMV-associated disease (18). Our results document the emergence of functional adaptive NKG2C^+^ NK cells in TAP-deficient patients. These NK cells have retained their ability to respond to agonistic signaling through NKG2C and mediate ADCC, thereby likely being able to contribute to antiviral immunity in TAP-deficient patients.

A relative weakness of the present study is the inability to specifically link the adaptive NK cell phenotypes observed to past CMV infection in the patients. Based on presently available literature, our presumption is that the TAP-deficient patients with expansion of NKG2C^+^ NK cells are CMV^+^. The prevalence of CMV infection in the human population is high, and the high frequencies of NKG2C^+^ NK cells in patients TAP#01 and TAP#02 were above the 99th percentile of those found in CMV^−^ healthy donors and mimicked those found in CMV^+^ donors (3). Notably, acute or latent infection with other herpes viruses has not been associated with expansion of NKG2C^+^ NK cells (35, 36). Although the current cohort includes 7 of the 33 cases with TAP deficiency described so far, it is still limited in size. However, expansion of NKG2C^+^ NK cells in three of the seven patients (42%) is in line with the frequencies of adaptive NK cell responses observed in large cohorts of healthy donors (3, 37). Unfortunately, there was no stored serum available to allow serological assessment of CMV status, and new sampling of the patients studied was not possible. In an alternative attempt to assess past CMV exposure, we performed CD8 T-cell assays with overlapping peptides from the CMV phosphoprotein pp65. Although we did observe a weak positive response in patient TAP#05, all other patients, including patient TAP#01 and TAP#02, were negative (data not shown). It is highly plausible that defective presentation of immunodominant epitopes in human TAP deficiency leads to weak or absent CD8 T-cell responses (38). Of note, in our previous experiments in a cohort of 204 healthy donors, all donors with evidence of adaptive NK cells were CMV seropositive and displayed robust T-cell responses to pp65 peptide stimulation (Ref. (3) and data not shown). We cannot formally exclude that other pathogenic challenges than CMV, or other inflammatory processes, triggered the expansion and differentiation of NKG2C^+^ NK cells in the studied patients. However, regardless of the causative agent, the data clearly demonstrate that TAP-deficient individuals are capable of expanding a population of NK cells with phenotypic features of adaptive NK cells. The present outcome with the emergence of adaptive NK cell responses in the absence of T-cell responses to viral antigens may reflect a biologically important scenario.

The cellular mechanisms involved in driving adaptive NK cell responses in the human are unknown. Phenotypic characterization of expanded NK cell populations suggests a major contribution of DAP-12-coupled activating receptors including NKG2C and activating KIRs (3, 9, 39). Coculture of NK cells with HCMV-infected fibroblasts leads to expansion of NKG2C^+^ NK cells, supporting a direct involvement of NKG2C in driving the response (40, 41). The finding of NKG2C^+^ adaptive NK cell populations in patients with TAP deficiency raised the question whether HLA-E expression levels were modulated by HCMV infection in TAP-deficient cells. We found that HCMV infection lead to a further decrease in overall levels of HLA class I without influencing HLA-E that was maintained at approximately 30–50% of that in normal individuals. HLA-E expression was previously reported to be sensitive to the CMV-encoded protein US6 glycoprotein but resistant to US2 and US11 (42). Since US6 exerts its function through TAP inhibition, it will not down-modulate HLA-E expression in TAP-deficient fibroblasts, potentially explaining that the levels of HLA-E remained unaltered in IE1^+^ fibroblasts. Alternatively, the relative stability of HLA-E expression levels may be due to stabilization by the UL40 leader-sequence peptide, which is TAP-independent (43). Although not formally shown, these data are compatible with the notion that HCMV-infected TAP-deficient cells may trigger NKG2C receptors and drive the expansion of adaptive NK cells in these patients. However, it is also possible that the expansion of NKG2C^+^ NK cells in TAP-deficient individuals is driven by CD16 and antibody-mediated recognition of viral antigens (44).

In contrast to adaptive NK cell responses in apparently healthy human blood donors, the expanding NKG2C^+^ NK cells in TAP-deficient patients displayed polyclonal KIR profiles. An important inference from this finding is that NK cell education, and the enhanced functional potential associated with this state, is not an absolute prerequisite for human adaptive NK cell responses to occur. This is in line with the data in MHC class I-deficient mice, which control MCMV infection equally well as wild-type mice and mount robust adaptive NK cell responses with expansion of Ly49H^+^ NK cells upon viral infection (14). These results support the notion that the skewing of KIR repertoires toward cells expressing self-specific inhibitory KIRs, commonly associated with CMV infection and CMV reactivation, could be a result of preferential survival of such subsets in conditions of stress, or in the context of excess stimulation with, e.g., IL-15 (12). Given the high level of enrichment of NKG2C^+^ NK cells expressing self-KIR in normal TAP-expressing donors, there is also the possibility that NK cells compete during the expansion phase, supported by the tendency of relatively lower degrees of differentiation of adaptive NK cells in TAP-deficient donors. Along the same lines, the skewing of the NK cell repertoire by CMV infection may depend on constitutive inhibitory signaling through KIR and its potential influence on cellular interactions with host cells (45).

Although this study is based on few patients with defined deficiency in the TAP-1/2 proteins, the findings clearly illustrate the expansion of polyclonal adaptive NK cells in the context of low HLA class I expression in the host. The insight that inhibitory KIR and education are not essential components of the upstream events in the human adaptive NK cell responses is informative and may be of relevance for understanding the cellular drivers of NK cell differentiation. Notably, in this context, adaptive NK cells in TAP-deficient patients respond weakly to stimulation of NKG2C and CD16, both suggested to be involved in driving proliferation of adaptive NK cells (41, 44). It is tempting to speculate that NKG2C^+^ NK cells contribute to the relatively low incidence of viral complications seen in TAP-deficient patients (18). Conversely, the possibility that these cells contribute to immune pathology and necrotizing granulomatous skin lesions may be considered.

## Author Contributions

VB, MS, H-GL, JZ, and KM designed research. VB, MS, JG, and MK performed research and analyzed data. JZ, JG, HR, H-GL edited the manuscript. VB and KM wrote the manuscript.

## Conflict of Interest Statement

The authors declare that the research was conducted in the absence of any commercial or financial relationships that could be construed as a potential conflict of interest.

## Supplementary Material

The Supplementary Material for this article can be found online at http://journal.frontiersin.org/article/10.3389/fimmu.2015.00507

## Funding

This work was supported by grants from the Swedish Children’s Cancer Society, the Swedish Cancer Society, the Swedish Research Council, the Royal Swedish Academy of Sciences, the Tobias Foundation, the Karolinska Institutet, the Wenner-Gren Foundation, the Norwegian Research Council, the KG Jebsen Foundation, the Norwegian Cancer Society, the Oslo University Hospital, and the National Research Fund of Luxembourg (FNR, AFR PhD grant to MS). VB is supported by the French National Research Agency (ANR) (grant no. NKIR-ANR-13-PDOC-0025-01).
