# Supplementary figures and images for: Polyclonal Expansion of NKG2C+ NK Cells in TAP-Deficient Patients
Source: Front Immunol. 2015 Oct 6;6:507. doi: 10.3389/fimmu.2015.00507 (PMC4594010; doi:10.3389/fimmu.2015.00507)

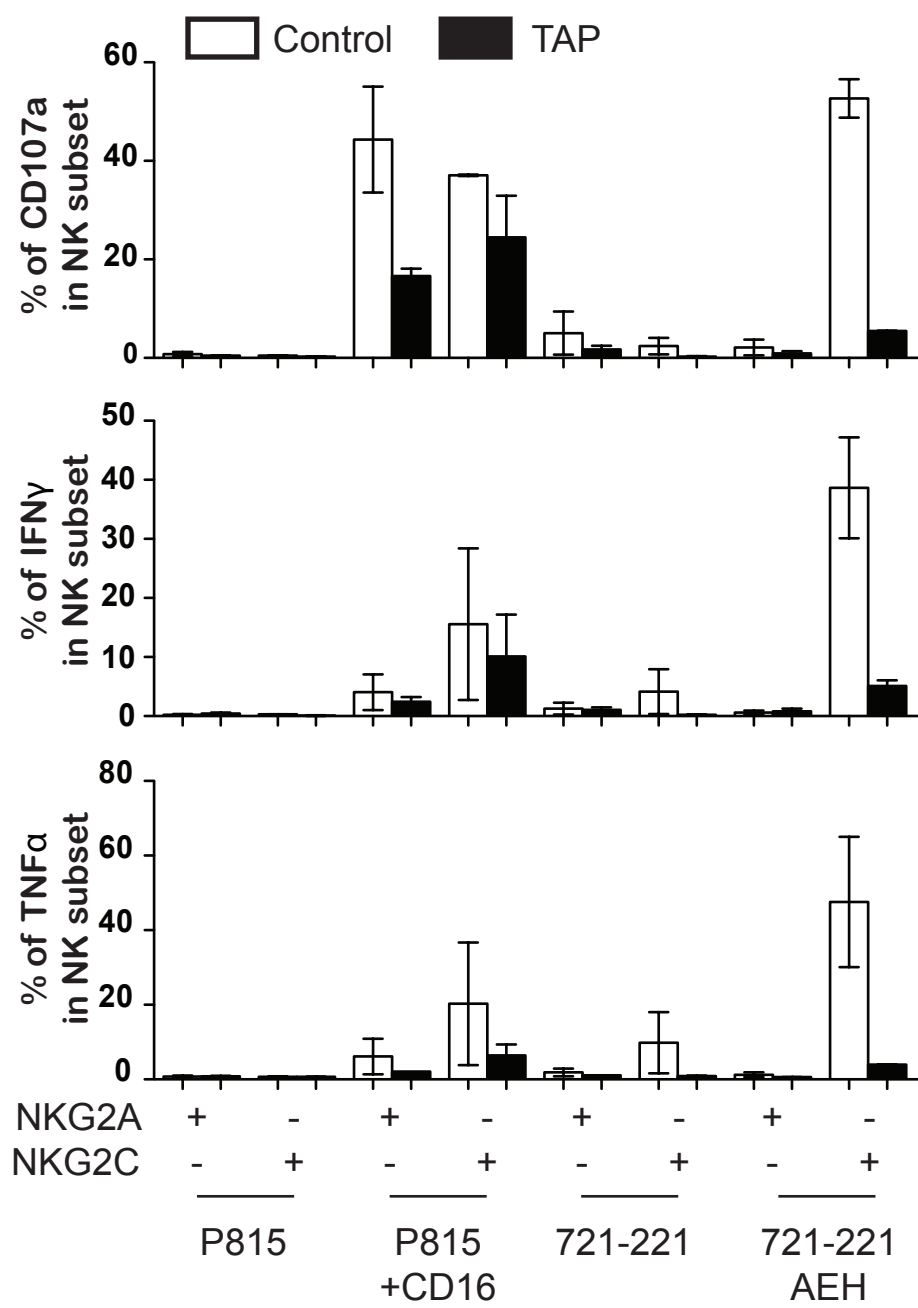

Supplement: Supplementary file 1 [file Image_1.PDF]
